# Integrated Analysis of Immunity- and Ferroptosis-Related Biomarker Signatures to Improve the Prognosis Prediction of Hepatocellular Carcinoma

**DOI:** 10.3389/fgene.2020.614888

**Published:** 2020-12-18

**Authors:** Xuanlong Du, Yewei Zhang

**Affiliations:** Department of General Surgery, Zhongda Hospital, School of Medicine, Southeast University, Nanjing, China

**Keywords:** hepatocellular carcinoma, ferroptosis, immunity, overall survival, biomarker

## Abstract

**Background:**

Hepatocellular carcinoma (HCC) is a common malignant tumor with high mortality and poor prognoses around the world. Ferroptosis is a new form of cell death, and some studies have found that it is related to cancer immunotherapy. The aim of our research was to find immunity- and ferroptosis-related biomarkers to improve the treatment and prognosis of HCC by bioinformatics analysis.

**Methods:**

First, we obtained the original RNA sequencing (RNA-seq) expression data and corresponding clinical data of HCC from The Cancer Genome Atlas (TGCA) database and performed differential analysis. Second, we used immunity- and ferroptosis-related differentially expressed genes (DEGs) to perform a computational difference algorithm and Cox regression analysis. Third, we explored the potential molecular mechanisms and properties of immunity- and ferroptosis-related DEGs by computational biology and performed a new prognostic index based on immunity- and ferroptosis-related DEGs by multivariable Cox analysis. Finally, we used HCC data from International Cancer Genome Consortium (ICGC) data to perform validation.

**Results:**

We obtained 31 immunity (*p* < 0.001)- and 14 ferroptosis (*p* < 0.05)-related DEGs correlated with overall survival (OS) in the univariate Cox regression analysis. Then, we screened five immunity- and two ferroptosis-related DEGs (HSPA4, ISG20L2, NRAS, IL17D, NDRG1, ACSL4, and G6PD) to establish a predictive model by multivariate Cox regression analysis. Receiver operating characteristic (ROC) and Kaplan–Meier (K–M) analyses demonstrated a good performance of the seven-biomarker signature. Functional enrichment analysis including Gene Ontology (GO) and the Kyoto Encyclopedia of Genes and Genomes (KEGG) revealed that the seven-biomarker signature was mainly associated with HCC-related biological processes such as nuclear division and the cell cycle, and the immune status was different between the two risk groups.

**Conclusion:**

Our results suggest that this specific seven-biomarker signature may be clinically useful in the prediction of HCC prognoses beyond conventional clinicopathological factors. Moreover, it also brings us new insights into the molecular mechanisms of HCC.

## Introduction

Liver cancer is a common malignant tumor around the world. Liver cancer is the seventh most common cancer globally according to global cancer data in 2017, with 953,000 new cases diagnosed and 819,000 deaths (Fitzmaurice et al., 2019). Hepatocellular carcinoma (HCC) is the most common because HCC accounts for approximately 90% of primary liver cancer (Llovet et al., 2016). Risk factors for HCC mainly include cirrhosis (chronic liver damage caused by inflammation and fibrosis), hepatitis B virus (HBV) infection, hepatitis C virus (HCV) infection, alcohol abuse, and non-alcoholic fatty liver disease (NAFLD) (Fujiwara et al., 2018). HCC is very malignant, and 70% of patients undergoing surgery experience tumor recurrence within 5 years (Nakagawa et al., 2016). In addition, HCC is highly complex and heterogeneous, so many molecular targeted anticancer agents are not effective for some patients, with some even showing resistance (Cancer Genome Atlas Research Network, 2017). The prognosis of HCC is very poor, with a 3 years survival rate of 12.7% and a 5 years survival rate of 20% (Giannini et al., 2015; Goossens et al., 2015; Yu et al., 2017). Currently, the diagnosis and treatment of HCC are not satisfactory. Specific biomarkers play an important role in the early screening, diagnosis, treatment option selection, and prognosis of HCC. In our study, we explored some immunity- and ferroptosis-related biomarkers to understand how they affect the pathogenesis and prognosis of HCC. We hope that these biomarkers will be helpful for the diagnosis, treatment, and prognosis of HCC.

Ferroptosis is an iron-dependent form of non-apoptotic cell death in the presence of oxidized polyunsaturated fatty acids (PUFAs), redox-active iron, and compromised lipid peroxide repair (Perez et al., 2020). Ferroptosis has been demonstrated in many diseases, such as cancer (Liang et al., 2019; Perez et al., 2020). It also plays a very vital role in digestive-system neoplasms such as gastric cancer, pancreatic cancer, colorectal cancer, and, especially, HCC (Nie et al., 2018). Research shows that the p62-Keap1-NRF2 pathway is related to the ferroptosis of liver cancer cells, and the retinoblastoma (RB) protein plays a role in liver tumorigenesis and ferroptosis (Viatour et al., 2011; Sun et al., 2016; Nie et al., 2018). Therefore, it is important to research ferroptosis-related biomarkers in the prognosis and treatment of HCC patients.

In total, 80% of HCC patients in an advanced stage lost opportunities for surgery and ablation, but systemic treatments for HCC are limited (Liu and Qin, 2019). In recent years, immunotherapy has been given more attention, and clinical trials and animal experiments have proven that immunotherapy plays a role in the treatment of HCC patients (Zongyi and Xiaowu, 2020). Immunotherapy approaches, including vaccines, immune checkpoint blockade, and adoptive cell transfer (ACT), have been proven safe and effective for HCC treatment (Li et al., 2015). Therefore, it is important to explore immunity-related biomarkers for immunotherapy treatment of HCC.

In summary, we find that ferroptosis in HCC must be related to immunity. Lang et al. (2019) found that radiotherapy and immunotherapy can promote ferroptosis *via* SLC7A11. Ubellacker et al. (2020) proved that metastasizing melanoma cells from the lymph nodes are resistant to ferroptosis. Jiang et al. (2020) designed sulfasalazine (SAS)−loaded mesoporous magnetic nanoparticles (Fe_3_O_4_) and platelet (PLT) membrane camouflage (Fe_3_O_4_-SAS @ PLT), which can mediate ferroptosis with immunotherapy and can be expected to provide great potential in the clinical treatment of tumor metastasis. Ruiz-de-Angulo et al. (2020) developed iron oxide-loaded nanovaccines (IONVs) that can enhance the combination immunotherapy and immunotherapy-promoted tumor ferroptosis. We find that exploring the clinical relevance and prognostic significance of immunity- and ferroptosis-related biomarkers is helpful for ferroptosis immunotherapy in HCC.

In this study, we used TCGA database to analyze the mRNA expression profiles to find immunity- and ferroptosis-related differentially expressed genes (DEGs) for the prognosis of HCC. Furthermore, functional analysis of potential immunity- and ferroptosis-related DEGs is helpful for understanding their roles in the occurrence and development of HCC. Finally, we also validated our results in the International Cancer Genome Consortium (ICGC) cohort. Therefore, this study provides a good prognostic risk model for HCC patients and some insights into the occurrence and development of HCC.

## Materials and Methods

### Patients and Datasets

The RNA sequencing (RNA-seq) data and corresponding clinical information of 374 HCC samples and 50 normal liver samples were downloaded from TCGA database^1^ on September 10, 2020. In addition, The RNA-seq data and corresponding clinical information of 231 liver cancer samples were obtained from the ICGC database^2^ on September 10, 2020. From the Immunology Database and Analysis Portal (ImmPort), a list of 2,483 immunity-related genes was obtained. ImmPort is a database that updates immunology data accurately and in a timely manner. Data from ImmPort are a powerful foundation of immunology research. In addition, it provides a list of immunity-related genes for use in cancer research. These genes were proven to participate in the process of immune activity. Additionally, 60 ferroptosis-related genes were obtained by summarizing previous literature.

### Differential Gene Analysis

By comparing HCC tissues to normal tissues and using the limma package from Bioconductor in R software (version 4.0.2), DEGs were identified with the criteria for screening the DEGs of a false discovery rate (FDR) < 0.05 and a | log2FoldChange| > 1. Then, immunity- and ferroptosis-related DEGs were extracted from the DEGs.

### Construction and Validation of the Prognostic Immunity- and Ferroptosis-Related Differentially Expressed Gene Signature

The association between immunity- and ferroptosis-related DEGs and patient survival was evaluated by univariate Cox regression analysis using the survival R package in R. Immunity-related DEGs with a *p* < 0.001 and ferroptosis-related DEGs with a *p* < 0.05 were considered candidate variables in a univariate Cox regression analysis and entered into a stepwise multivariate Cox regression analysis tested by Akaike Information Criterion (AIC, assessing the goodness of fit of a statistical model) to identify covariates with independent prognostic values for patient survival. Based on the median risk score, HCC patients were divided into high- and low-risk groups. The Kaplan–Meier (K–M) survival curves for cases predicted with low and high risk were generated, respectively. Then, ROC curve analysis was performed to test the sensitivity and specificity of the prognostic risk score model for overall survival (OS) in HCC patients. The area under the ROC curve (AUC) was derived as reported previously. Then, we performed an independent prognostic analysis to evaluate whether clinical parameters are independent prognostic factors for OS. Based on prognostic immunity-and ferroptosis-related DEGs, we performed principal component analysis (PCA) by the “prcomp” function of the “stats” R package and used t-distributed stochastic neighbor embedding (t-SNE) with the “Rtsne” R package. To verify these results, we used ICGC data.

### Construction of the Immunity- and Ferroptosis-Related Differentially Expressed Gene Regulatory and Protein–Protein Interaction Network

We explored prognostic immunity- and ferroptosis-related DEG regulatory mechanisms, extracted prognostic immunity-related DEGs to construct the regulatory network of immunity- and ferroptosis-related DEGs, and used Cytoscape software (version 3.7.2) to display the immunity- and ferroptosis-related DEG regulatory network. To explore the interactions between these genes, we constructed a protein–protein interaction (PPI) network based on data gleaned from the Retrieval of Interacting Genes (STRING) online database^3^.

### Functional Enrichment Analysis

Based on the median risk score, HCC patients were divided into the high-and low-risk groups. We applied the “limma” R package to analyze the correlations of DEGs between the high- and low-risk groups with FDR < 0.05 and | log2FoldChange| > 1 in TCGA and ICGC cohorts, respectively. Then, we used risk-related DEGs to conduct Gene Ontology (GO) and Kyoto Encyclopedia of Genes and Genomes (KEGG) analyses by the “clusterProfiler” R package. Finally, for differential infiltrating score analysis between the high- and low-risk groups, we determined the infiltrating scores of immune cells and immune-related functions for samples by single-sample gene set enrichment analysis (ssGSEA).

## Results

### Identification of Immunity- and Ferroptosis-Related Genes

**FIGURE 1:**
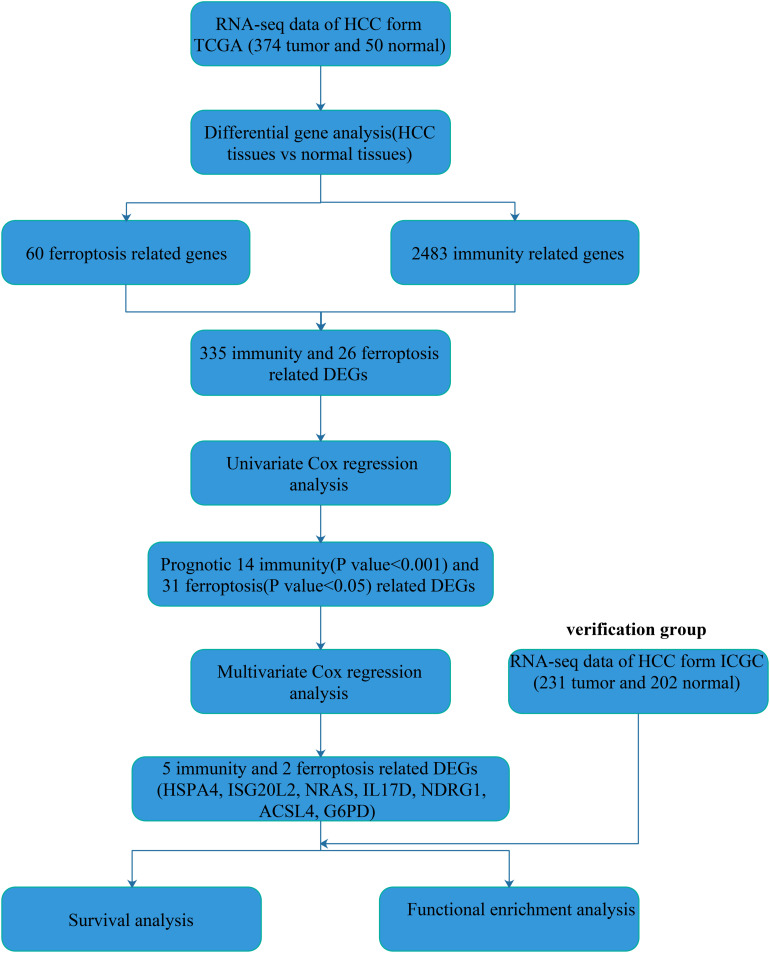
Flowchart of our study.

Using the limma package of R language for DEGs (FDR < 0.05 and | log2FoldChange | > 1) analysis, we obtained 7,768 DEGs in the 374 HCC samples and 50 normal liver samples from TCGA database. Then, we extracted 335 immunity-related DEGs from 2,483 entries of the ImmPort database and 26 ferroptosis-related DEGs from 60 ferroptosis-related genes in literature reporting (Table 1). The flow diagram of this study is shown (Figure 1).

**TABLE 1:** Sixty ferroptosis-related genes.

**ACSL4**	**ALOX12**	**CISD1**	**GLS2**	**LPCAT3**	**SLC7A11**	**PHKG2**	**ACSL3**	**NFE2L2**	**GOT1**
AKR1C1	ATP5MC3	CS	GPX4	MT1G	FDFT1	HSBP1	ACACA	KEAP1	G6PD
AKR1C2	CARS1	DPP4	GSS	NCOA4	TFRC	ACO1	PEBP1	NQO1	PGD
AKR1C3	CBS	FANCD2	HMGCR	PTGS2	TP53	FTH1	ZEB1	NOX1	IREB2
ALOX15	CD44	GCLC	HSPB1	RPL8	EMC2	STEAP3	SQLE	ABCC1	HMOX1
ALOX5	CHAC1	GCLM	CRYAB	SAT1	AIFM2	NFS1	FADS2	SLC1A5	ACSF2

### Identification of Prognostic Immunity- and Ferroptosis-Related Differentially Expressed Genes

**FIGURE 2:**
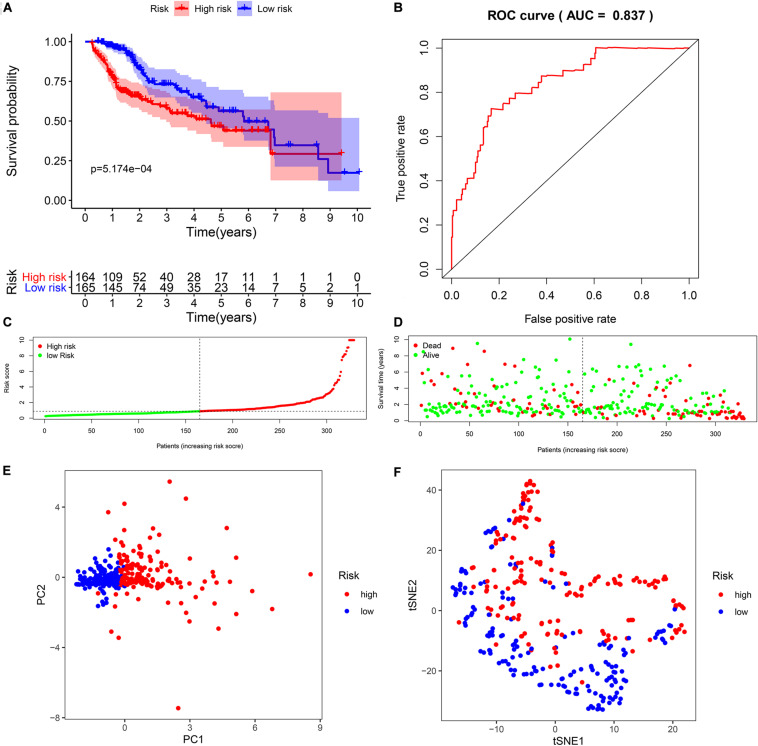
Survival analysis of seven biomarkers in The Cancer Genome Atlas (TCGA) cohort. **(A)** Kaplan–Meier survival curves for the seven biomarkers relative to the overall survival outcomes. **(B)** Receiver operating characteristic (ROC) analysis of the sensitivity and specificity of the survival time using the seven biomarkers based on the risk score. **(C)** Distribution of hepatocellular carcinoma (HCC) sample risk scores. Samples were divided into two groups based on the median risk score. **(D)** Survival status of HCC patients. The positions of the dots represent the correlations between the survival times and risk scores. **(E)** Principal component analysis (PCA) plot of TCGA cohort. **(F)** T-distributed stochastic neighbor embedding (t-SNE) analysis of TCGA cohort.

To explore the immunity- and ferroptosis-related DEG correlations with the OS of HCC patients, we obtained 31 immunity (*p* < 0.001)- and 14 ferroptosis (*p* < 0.05)-related DEGs by univariate Cox regression analysis (Tables 2, 3). According to the forest plot of hazard ratios (HRs), most of these DEGs were risk factors for poor prognoses in HCC patients. Then, we used these DEGs to perform multivariate Cox regression analysis. Finally, we identified five immunity- and two ferroptosis-related DEGs (HSPA4, ISG20L2, NRAS, IL17D, NDRG1, ACSL4, and G6PD) to establish a predictive model (Table 4). K-M survival curves outcomes based on median risk score values show that the predicted survival time of the low-risk group was significantly longer than that of the high-risk group, *p* < 0.001 (Figure 2A). We used the time-dependent ROC curve to assess the ability of the risk score to predict survival rates (Figure 2B). The results showed that the AUC of the five immunity- and two ferroptosis-related DEGs in the prognostic model was 0.837 and demonstrated that the risk score model had a stable performance. HCC patients were classified into high- and low-risk groups according to respective median risk scores (Figures 2C,D). By PCA and t-SNE analyses, we also indicated that HCC patients in different risk groups were distributed in two directions (Figures 2E,F).

**TABLE 2:** Univariate Cox regression analysis of 31 immunity (*p* < 0.001)-related DEGs.

**Gene**	**HR**	**HR.95L**	**HR.95H**	***p*-value**
HSPA4	1.045547	1.026417	1.065033	2.27303e–06
HSP90AA1	1.004311	1.002005	1.006623	0.000244
PSMD2	1.020485	1.009187	1.031909	0.000356
PSMD10	1.051077	1.022360	1.080600	0.000424
PSME3	1.045486	1.018561	1.073124	0.000833
PSMD14	1.108899	1.068529	1.150793	4.677158e–08
IFI30	2.120626	1.388905	3.237842	0.000498
S100A10	1.003209	1.001529	1.004892	0.000178
S100A11	1.001503	1.000696	1.002310	0.000257
FABP6	1.107941	1.051312	1.167619	0.000128
ISG20L2	1.150664	1.091781	1.212722	1.637690e–07
PPIA	1.012916	1.007300	1.018564	6.063610e–06
CACYBP	1.063116	1.041823	1.084844	3.045023e–09
TRAF3	1.314769	1.141606	1.514198	0.000145
DCK	1.128434	1.061451	1.199643	0.000108
EED	1.371412	1.139318	1.650788	0.000841
NDRG1	1.007402	1.004143	1.010673	8.191811e–06
HDAC1	1.043026	1.026902	1.059404	1.160481e–07
BIRC5	1.029163	1.015326	1.043189	3.150455e–05
NRAS	1.074759	1.045659	1.104669	2.632364e–07
CKLF	1.049034	1.020240	1.078640	0.000748
PLXNA1	1.136348	1.054735	1.224277	0.000775
PLXNA3	1.237200	1.109210	1.379960	0.000133
GMFB	1.127773	1.058756	1.201289	0.000189
IL17D	1.102332	1.049880	1.157405	8.969548e–05
KITLG	1.216591	1.091701	1.355768	0.000388
STC2	1.035485	1.0157342	1.055619	0.000386
BRD8	1.153320	1.062828	1.251517	0.000622
NR6A1	1.299958	1.123685	1.503884	0.000417
SHC1	1.014026	1.007082	1.021018	7.091489e–05
CDK4	1.030991	1.014361	1.047895	0.000234

**DEGs, differentially expressed genes; HR, hazard ratio.**

**TABLE 3:** Univariate Cox regression analysis of 14 ferroptosis (*p* < 0.05)-related DEGs.

**Gene**	**HR**	**HR.95L**	**HR.95H**	***p*-value**
ACSL4	1.002989	1.000515	1.005469	0.017831
AKR1C1	1.002368	1.000481	1.004259	0.0138674
AKR1C3	1.003731	1.001673	1.005794	0.000376
FANCD2	1.393143	1.179111	1.646025	9.781021e–05
SLC7A11	1.096822	1.030426	1.167497	0.003722
TFRC	1.024294	1.005358	1.043587	0.011694
AIFM2	1.047483	1.014825	1.081192	0.0040961
FTH1	1.001395	1.000482	1.002308	0.002725
STEAP3	0.987559	0.976359	0.998888	0.031473
ACACA	1.154423	1.068485	1.247272	0.000274
NQO1	1.002206	1.001001	1.003413	0.000330
ABCC1	1.083417	1.042739	1.125683	4.072212e–05
SLC1A5	1.014168	1.0076400	1.020738	1.955653e–05
G6PD	1.014428	1.010206	1.018668	1.6770876e–11

**DEGs, differentially expressed genes; HR, hazard ratio.**

**TABLE 4:** Multivariate Cox regression analysis of seven biomarkers.

**Gene**	**coef**	**HR**	**HR.95L**	**HR.95H**	***p*-value**
HSPA4	0.03010367	1.03056137	1.00997931	1.05156286	0.00344803
ISG20L2	0.07391033	1.07671025	1.01047236	1.14729014	0.02251551
NRAS	0.03852136	1.03927293	1.00807902	1.07143210	0.01323166
IL17D	0.07267629	1.07538237	1.01820693	1.13576838	0.00912692
ACSL4	0.00250016	1.00250329	0.99991487	1.00509840	0.05803585
G6PD	0.00752260	1.00755097	1.00139737	1.01374238	0.01609678
NDRG1	0.004278187	1.004287352	0.999529763	1.009067586	0.077424807

**coef, coefficient; HR, hazard ratio.**

### Construction of the Immunity- and Ferroptosis-Related Differentially Expressed Gene Regulatory and Protein–Protein Interaction Network

**FIGURE 3:**
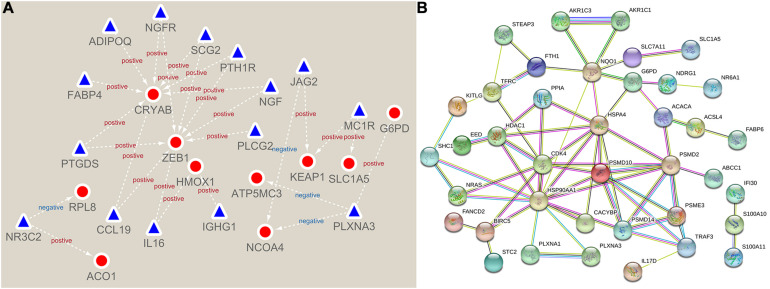
Network of immunity- and ferroptosis-related differentially expressed genes (DEGs). **(A)** The prognostic immunity- and ferroptosis-related DEG regulatory network. The red circles represent ferroptosis-related DEGs, and the blue triangles represent immunity-related DEGs. **(B)** The protein–protein interaction (PPI) network of prognostic immunity- and ferroptosis-related DEGs.

First, we constructed a regulatory network about prognostic 31 immunity- and 14 ferroptosis-related DEGs (Figure 3A). Then, using the STRING online platform, we established a PPI network based on these DEGs (Figure 3B). Both networks indicated that immunity and ferroptosis have potential molecular mechanisms.

### Validation of the Seven-Biomarker Signature Using the International Cancer Genome Consortium Data

**FIGURE 4:**
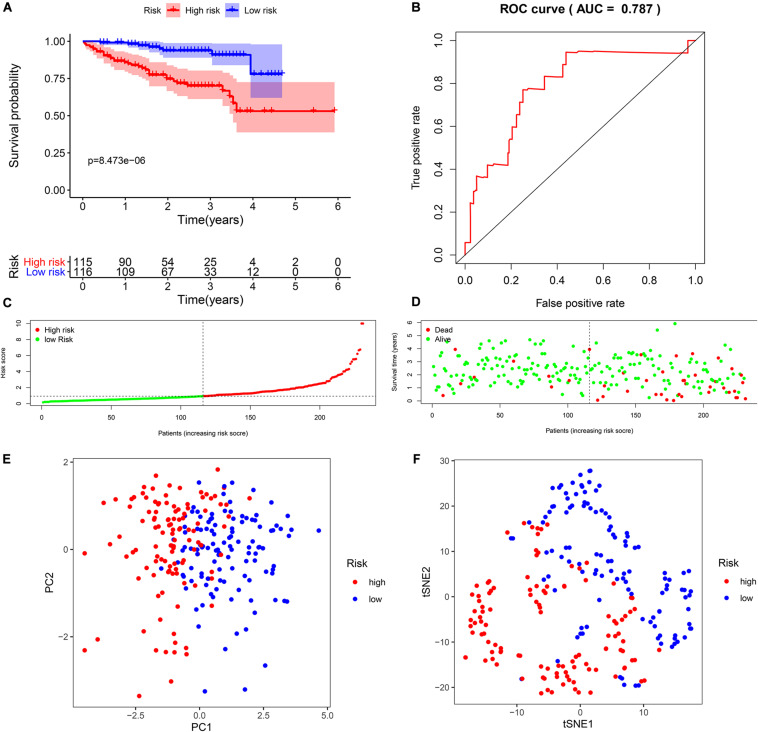
Validation of seven biomarkers in the International Cancer Genome Consortium (ICGC) cohort. **(A)** Kaplan–Meier survival curves for the seven biomarkers relative to the overall survival outcomes. **(B)** Receiver operating characteristic (ROC) analysis for the seven biomarkers based on risk scores. **(C)** The distribution and median value of the risk scores. **(D)** Survival statuses of hepatocellular carcinoma (HCC) patients. The positions of the dots represent the correlations between the survival time and risk scores. **(E)** Principal component analysis (PCA) plot in the ICGC cohort. **(F)** t-distributed stochastic neighbor embedding (t-SNE) analysis in the ICGC cohort.

To verify the reliability of the model from TCGA data, we selected seven biomarkers to perform multivariate Cox regression analysis using ICGC data. Likewise, compared to the median risk score values, the K-M survival curve outcomes show similar results (Figure 4A). In addition, the time-dependent ROC curve showed that the AUCs of the seven-biomarker prognostic model was 0.787 (Figure 4B). It also indicated that the risk score model was very robust. HCC patients with ICGC data were also categorized into high- and low-risk groups according to the respective median risk scores (Figures 4C,D). PCA and t-SNE analyses demonstrated that patients in the two groups were distributed in discrete directions (Figures 4E,F).

### Independent Prognostic Analysis

**FIGURE 5:**
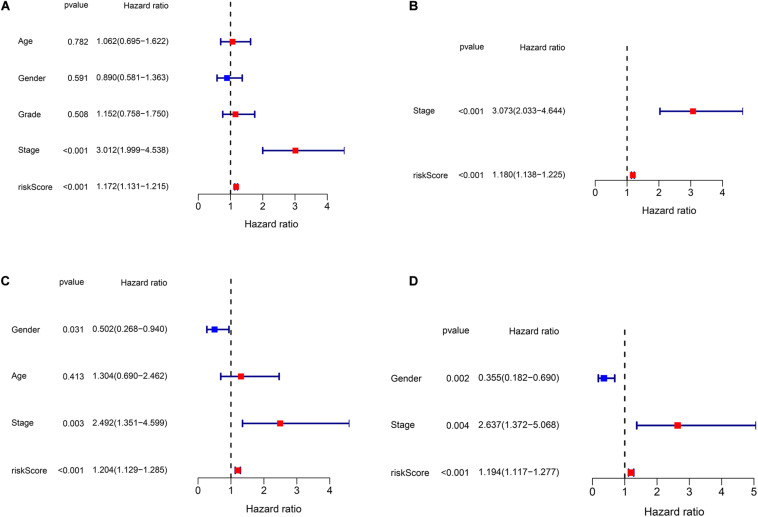
Independent prognostic analysis of risk scores and clinical parameters. **(A)** The univariate Cox regression analysis of the associations between the risk scores and clinical parameters and the overall survival (OS) of patients in The Cancer Genome Atlas (TCGA) cohort. **(B)** The multivariate Cox regression analysis of the associations between the risk scores and clinical parameters and the OS of patients in TCGA cohort. **(C)** The univariate Cox regression analysis of the associations between the risk scores and clinical parameters and the OS of patients in the ICGC cohort. **(D)** The multivariate Cox regression analysis of the associations between the risk scores and clinical parameters and the OS of patients in the ICGC cohort.

We performed univariate and multivariate Cox regression analyses to evaluate whether clinical parameters (including gender, age, stage, and grade) and the risk score are independent prognostic factors of OS. Then, in both TCGA and ICGC data, we found that the risk score and stage were independent prognostic predictors for OS in the univariate and multivariate Cox regression analyses (Figures 5A–D).

### Functional Enrichment Analyses

**FIGURE 6:**
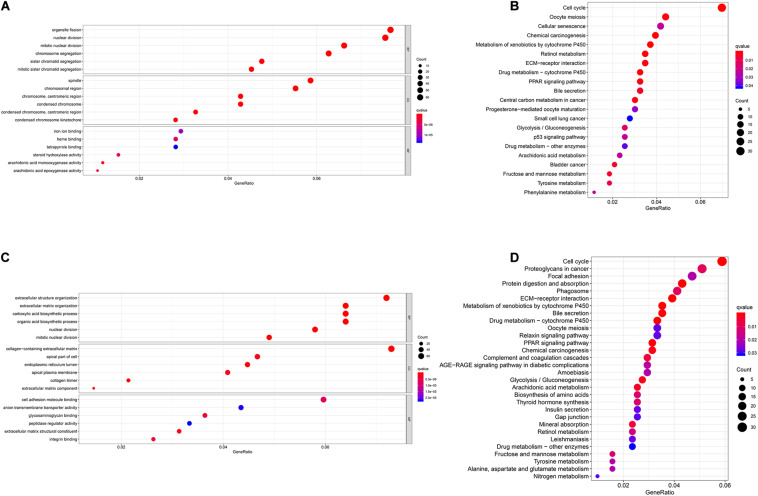
Gene Ontology (GO) and Kyoto Encyclopedia of Genes and Genomes (KEGG) enrichment analysis of seven biomarkers in The Cancer Genome Atlas (TCGA) and International Cancer Genome Consortium (ICGC) cohorts. **(A)** GO enrichment analysis of the seven biomarkers in TCGA cohort. **(B)** KEGG enrichment analysis of the seven biomarkers in TCGA cohort. **(C)** GO enrichment analysis of the seven biomarkers in the ICGC cohort. **(D)** KEGG enrichment analysis of the seven biomarkers in the ICGC cohort.

**FIGURE 7:**
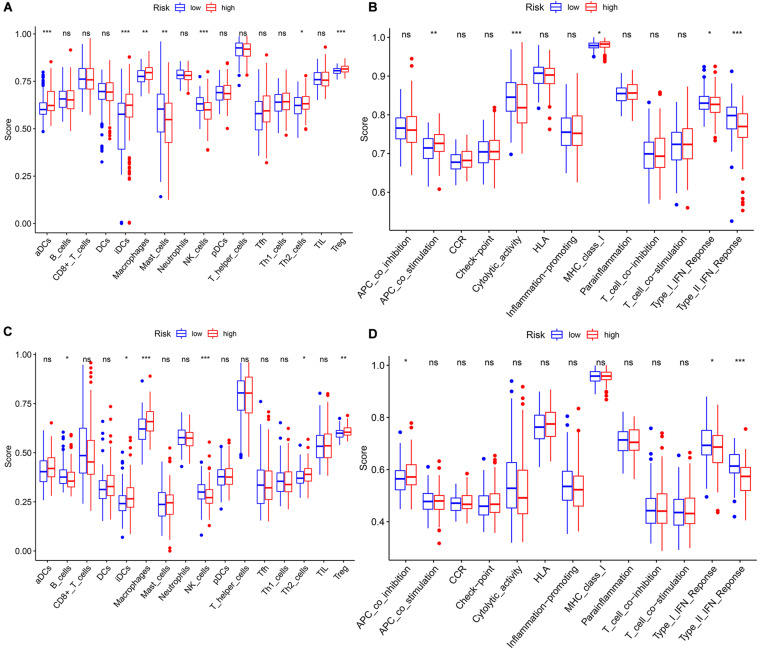
The single-sample gene set enrichment analysis (ssGSEA) scores between different risk groups in The Cancer Genome Atlas (TCGA) and International Cancer Genome Consortium (ICGC) cohorts. **(A)** Boxplots of the correlations between the scores of 16 immune cells and risk groups in TCGA cohort. **(B)** Boxplots of the correlations between 13 immune-related functions and risk groups in TCGA cohort. **(C)** Boxplots of the correlations between the scores of 16 immune cells and risk groups in the ICGC cohort. **(D)** Boxplots of the correlations of 13 immune-related functions and risk groups in the ICGC cohort. Adjusted *p*-values are shown as: ns, not significant; *p < 0.05; **p < 0.01; ***p < 0.001.

We performed GO and KEGG functional enrichment analyses on risk-related DEGs to investigate the potential functions of the seven prognostic biomarkers. The results indicated that the seven prognostic biomarkers mainly focused on nuclear division and mitotic nuclear division in both TCGA and ICGC data (Figures 6A,B). KEGG functional enrichment analysis suggested that the seven prognostic biomarkers were mainly related to the cell cycle, the metabolism of xenobiotics by cytochrome P450, extracellular matrix (ECM)–receptor interactions, etc. (Figures 6C,D). To further explore relationship between the HCC prognosis and immune status, we quantified the infiltrating scores of immune cell- and immunity-related functions with ssGSEA. The correlations between ssGSEA scores and different risk groups showed that the scores of iDCs, Macrophages, NK-cells, Th2-cells, Treg, APC costimulation, Type I IFN Response, and Type II IFN Response were significantly different between the low- and high-risk groups in both TCGA and ICGC cohorts (Figures 7A–D).

## Discussion

Although the current treatment and diagnosis of HCC have improved compared with the past, the mortality and incidence of HCC are still high. Colorectal cancer, stomach cancer, and HCC have the highest mortality rates, which have surpassed those of lung cancer (Chi et al., 2019). The prognosis is poor. It is very important to predict the prognosis of HCC and give corresponding treatments in time. The OS rate of HCC is still very low, and the prognosis is poor. The quality of life with advanced HCC is also poor (Anwanwan et al., 2020). Therefore, it is important to have deeper insight into the pathological mechanisms of the occurrence and development of HCC. In addition, it is vital to find new biomarkers and targets that are more sensitive and effective for the early diagnosis, treatment, and prognosis of HCC.

At present, many researchers have proven that ferroptosis is related to immunity, and immunotherapy and ferroptosis are both current research hotspots. However, it is rare to construct prognostic models of immunity- and ferroptosis-related genes and to predict their correlations in HCC. In our study, we systematically investigated the differential expression of immunity- and ferroptosis-related genes in HCC and performed a survival analysis. A novel prognostic model integrating five immunity- and two ferroptosis-related DEGs was first constructed and validated in an external cohort. Functional analyses show that immune cells and immunity-related functions and pathways were enriched.

We established the predictive model including five immunity- and two ferroptosis-related DEGs (HSPA4, ISG20L2, NRAS, IL17D, NDRG1, ACSL4, and G6PD). At present, the full name of HSPA4/Hsp70 is Heat Shock Protein Family A Member 4, which is a protein-coding gene. Chen et al. (2009) showed that the FOLFOX4 (5-fluorouracil, leucovorin, and oxaliplatin) regimen has advantages in colorectal cancer patients who have unresectable liver metastasis with lower HSP70 expression over those with higher HSP70 expression. Zhu et al. (2019) proved that ISG20L2 was related to the HCC prognosis. Concerning NRAS, Dietrich et al. (2019) found that it is a prognostic biomarker and contributes to sorafenib resistance in HCC. O’Sullivan et al. (2014) proved that IL17D was a novel target for the immunotherapy of tumors. NDRG1 is a hypoxia-inducible protein, which is related to the progression of human cancers, and is induced by hypoxia in HCCs (Sibold et al., 2007). Long non-coding RNA CCAT2 promotes proliferation and metastasis through upregulation of NDRG1 in HCC (Liu et al., 2019). The FOXQ1/NDRG1 axis can drive HCC initiation; thus, NDRG1 was suggested as a new potential therapeutic target for HCC (Luo et al., 2018). ACSL4 was overexpressed and served as an independent adverse prognostic index in HCC (Sun and Xu, 2017). Lu et al. (2018) found that overexpression of G6PD was correlated with epithelial–mesenchymal transition, which contributes to the migration and invasion of HCC.

At present, cancer treatment has entered an age of immunity and iron (Buttner et al., 2016; Tarangelo and Dixon, 2016). For ferroptosis, nanoparticles modulate iron and ROS levels to induce ferroptosis, which could provide a new strategy for cancer treatment (Tarangelo and Dixon, 2016). Immunotherapy has become a new standard of treatment for advanced HCC around the world (Waidmann, 2018). CD8^+^ T cells release interferon (IFN)γ, and IFNγ can regulate the lipid peroxidation and ferroptosis pathways in tumors (Wang et al., 2019). These findings mean that ferroptosis can play a role in the immunotherapy of tumors.

There are several limitations to our study. First, the number of normal samples from TCGA database was relatively small. Second, the present study only included a database mining design, without validation using fresh samples and prospective experimental research. Therefore, we will collect fresh samples and further prove our conclusions through experiments.

## Conclusion

In conclusion, we identified seven biomarkers associated with the prognosis of HCC patients. The prediction of the seven biomarkers’ functions provided further insight into the roles of the seven biomarkers in the immunotherapy and ferroptosis therapy of HCC. We also proved that the seven-biomarker signature can well predict the survival times of HCC patients. This signature has many potential prognostic and therapeutic implications for HCC patient management.

## Data Availability Statement

Publicly available datasets were analyzed in this study. This data can be found here: http://portal.gdc.cancer.gov/repository.

## Author Contributions

YZ conceived and designed the study. XD conducted the analysis and wrote the manuscript. Both authors read and approved the final manuscript.

## Conflict of Interest

The authors declare that the research was conducted in the absence of any commercial or financial relationships that could be construed as a potential conflict of interest.

## Footnotes

1 http://portal.gdc.cancer.gov/repository

2 https://dcc.icgc.org/projects/LIRI-JP

3 https://stringdb.org/
